# Soil nutrient loss through erosion: Impact of different cropping systems and soil amendments in Ghana

**DOI:** 10.1371/journal.pone.0208250

**Published:** 2018-12-19

**Authors:** Janvier Bigabwa Bashagaluke, Vincent Logah, Andrews Opoku, Joseph Sarkodie-Addo, Charles Quansah

**Affiliations:** 1 Department of Crop & Soil Sciences, Kwame Nkrumah University of Science and Technology (KNUST), Kumasi, Ghana; 2 Faculty of Agronomy, Université Catholique de Bukavu (UCB), Bukavu, Democratic Republic of Congo; 3 Institut Supérieur de Techniques de Développement, ISTD, Kalehe, Democratic Republic of Congo; Massachusetts Institute of Technology, UNITED STATES

## Abstract

Soil erosion is a multifactor threat to crop production and the environment. Most studies on soil erosion characterization have not focused on soil nutrient loss associated with erosion. The aim of this study was therefore to quantify the magnitude of nutrient loss through soil erosion under different cropping systems and amendments to inform agronomic practices in sub-Saharan Africa (SSA). A field experiment was carried out on runoff plots with different cropping systems (sole maize, sole cowpea, sole maize and maize intercropped with soybean) as main plots and soil amendments (biochar, NPK (Nitrogen +Phosphorus +Potassium) fertilizer, NPK + biochar and a control (no amendment)) constituting the subplots in a randomized complete block design. For each block, a bare plot was included to assess the efficiency of the different crop and soil management practices on soil erosion. The study was carried out in three consecutive cropping seasons in the semi-deciduous forest zone of Ghana. The bare plots had the highest amounts of nitrogen (N), phosphorus (P), and potassium (K) eroded: 33.88, 12.35 and 12.75 kg ha^-1^ respectively followed by the control plots with magnitude of 20.43, 8.42 and 7.87 kg ha^-1^ respectively for N, P and K. Sole maize had the highest amounts of nutrient loss: 19.71, 8.12 and 7.27 for N, P and K respectively compared to all the other cropping systems where the losses varied respectively from 12.38 to 17.12, 6.67 to 7.49 and 5.81 to 6.75 kg ha^-1^ The legume-based cropping systems under inorganic fertilizer and biochar management effectively reduced nutrient loss more than all other treatment combinations. The off-site effect of soil erosion expressed as enrichment ratio (ER) was higher for all plots, which received inorganic fertilizer inputs varying from 1.93 to 3.06 while the other treatments had ERs of 1.51 to 2.03. The ERs of fine soil particles were greater than 1 (ranging from 1.14 to 3.6) being relatively higher than that of coarse particles (sand) with values below 1 (ranging from 0.62 to 0.88). The least cumulative monetary value of nutrient loss (30.82 US$ ha^-1^) was observed under cowpea cropping system which received NPK + BC treatment. Soil erosion affected directly soil nutrient depletion through nutrient loss; however, integrated soil fertility management associated with legume-based cropping systems can be alternative options to reducing its effects on croplands in SSA.

## 1. Introduction

Soil erosion reduces the agricultural value of lands via physico-chemical degradations. Soil nutrient loss through runoff and sediment,is a major driver for soil fertility decline [1,2]. The eroded sediments or soil are highly concentrated with crop nutrients, which are washed away from farmlands. Erosion-based constraints coupled with unfavorable climatic conditions define significantly the productivity of farming systems in sub-Saharan Africa (SSA). Soil erosion leads to extreme losses of economic and environmental resources which negatively impact the economies of affected regions [3,4]. On-site consequences are directly observed on crop production as well as soil properties, affecting adversely the ability of the soil to respond to management practices with time. The amount of nutrients transported from croplands depends on the available soil management practices and the type of farming system.

The nutrients transported through plant harvest (yield and crop residues) coupled with nutrient loss through erosion (runoff and sediment) are important threats to soil nutrient depletion in SSA and defines the state of soils within the region. As a result, most soils in SSA are highly degraded such that specific integrated management practices involving organic and inorganic amendments are required.

Biochar is a carbon rich, relatively stable organic compound produced from the pyrolysis of biomass-derived feedstocks [5]. Due to its positive effects on soil properties, crop productivity and environment protection, biochar is being promoted and integrated into soil management systems [6]. However, its effect on soil erosion under cropping systems is limitedly studied in SSA. In this study, we hypothesized that biochar–crop interaction will reduce soil and nutrient losses from arable lands in SSA.

The nutrients lost to soil erosion process can be expressed economically to reflect the impact of erosion on fertilizer investment. The loss of soil nutrients through erosion indicates significant cost because of the need for replacement to enhance sustainability of cropping systems. In small-scale farming systems, this cost is not considered due to lack of relevant information [7]. Thus, its quantification can help different stakeholders to adopt the most effective soil and crop management practices to reduce loss and improve crop productivity [8]. From their study, [9] found that the seasonal cost of N, P and K lost through erosion under a maize monocrop grown under excessively tilled land was US$ 7.1 per hectare. According to [10], globally the estimated cost of land degradation ranges from 1.1 to 2.4% of Gross Domestic Product (GDP), corresponding to 2.9 to 6.3% of Agricultural Gross Domestic Product (AGDP). Beside the different nutrient losses, soil erosion affects the ecosystem via nutrient deposition and sedimentation and which is characterized by high enrichment ratios. During the erosion process, the different soil particles have different capacities to be transported based on their densities. For developing countries in SSA, whose economies depend heavily on the agricultural sector, the loss of agricultural productivity particularly through erosion, implies loss of revenue for the socio-economic development [11]. However, only few studies are devoted to the economic implications of soil fertility erosion under different cropping systems and fertility management practices [12] compared to other soil erosion characteristics such as sediment and runoff [7]. To bridge this gap in knowledge, nutrient loss via sediments and runoff pathways of erosion were studied under selected cropping systems in SSA. The aim of the study was to quantify soil nutrient loss and the associated costs due to erosion under specific crop and soil management practices.

## 2. Materials and methods

### 2.1. Research area description

The field experiment was carried out at the Faculty of Agriculture Research Station of the Kwame Nkrumah University of Science and Technology, at Anwomaso, Kumasi, Ghana. The site is located within the semi-deciduous forest zone of Ghana and lies on longitude 1.525° W and latitude 6.697° N. In this zone, farmers predominantly cultivate maize, cowpea, cassava essentially on subsistence basis. The experimental field was a one–year fallow land, which hitherto, was used for maize production under different tillage and soil management practices. The natural vegetation was dominated by guinea grass during the fallow period.

The zone is characterized by two cropping seasons: March to August as the major season and September to December as the minor season as a result of bimodal rainfall regime. The annual rainfall amount of the semi—deciduous forest zone of Ghana ranges from 1300 and 1500 mm [13,14]. However, during the research period, the total rainfall amounts received at the experimental site were 387, 272 and 466 mm during the 2016-major, 2016-minor and 2017-major seasons, respectively. The mean monthly temperature ranged between 24 and 28° C and the soil type, according to the World Reference Base (WRB) classification is Nitisol [15]. The slope of the experiment field varied from 3 to 10%. The experiment site was characterized by a moderate acidity (pH = 5.66 ± 0.013). Soil organic carbon and total nitrogen contents were 1.20 ± 0.02% and 0.09 ± 0.001% respectively. Available phosphorus, exchangeable potassium and CEC were 15.67 ± 0.083 mg kg^-1^, 0.02 ± 0.001 cmol_c_ kg^-1^ and 8 ± 0.040 cmol_c_ kg^-1^, respectively.

### 2.2. Study establishment and management

The experiment comprised of two factors: cropping systems (Maize + soybean intercrop, sole maize, sole soybean, and sole cowpea) and soil amendments (Control, NPK, NPK+ Biochar and Sole Biochar). Overall, the layout was a split–plot arranged in a randomized complete block design (RCBD) with cropping systems as main plot factor and soil amendments designated as sub-plot. The rates of each soil amendment varied with the crops cultivated and were 90-60-60 kg ha^-1^; 20-40-20 kg ha^-1^; 20-40-20 kg ha^-1^ of N, P_2_O_5_ and K_2_O for maize, soybean and cowpea respectively [16] and 5 t ha^-1^ of biochar [17] while for the combination of the two amendments (NPK+BC), 50% NPK and 50% BC were applied. Straight inorganic fertilizers (Urea, TSP and KCl) were applied as sources of nutrients (N, P and K) based on the recommended rates for each crop. The depth of application was approximately 5 cm. Fertilizers were applied two weeks after sowing; with split application of urea for maize: 2/3 of the rate was applied two weeks after sowing and the 1/3 remaining, four weeks after sowing. The biochar used in the study was produced from rice husk pyrolyzed at 500–600°C. The applied biochar had an alkaline pH (8.77) and total N, P and K contents of 0.56%, 0.67% and 0.52% respectively. Its C/N ratio was 68 whilst the ash content was 47.12%.

Each treatment was replicated three times. Each block had 16 plots under cultivation for the 16 treatment combinations (4 x 4) plus 1 bare plot as erosion check or control. Every individual plot measured 12 m x 3 m separated from the following plot with aluzinc sheets fixed 0.5 m deep to protect again the wind and 0.75 m of height at the surface to avoid any runoff contamination from the neighbouring plots. The observations on soil nutrient loss were carried out during three consecutive growing seasons (2016-major, 2016-minor and 207-major) and the field was under natural rainfall regime.

### 2.3. Runoff and sediment measurement with tipping buckets

**Fig 1:**
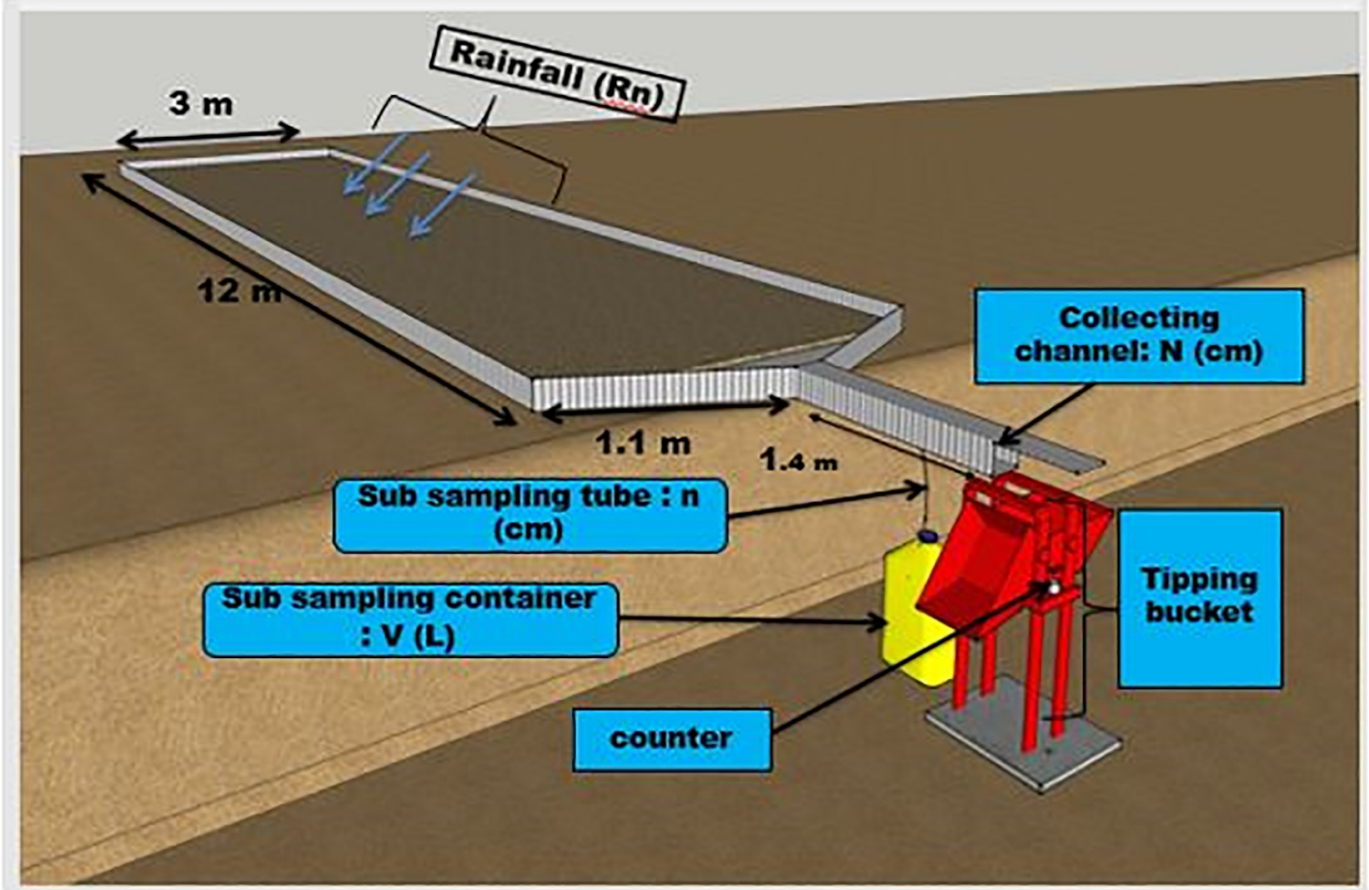
Layout of runoff plot with the tipping bucket device for runoff and soil erosion assessment.

The runoff amount from the plots was collected at the base of each runoff plot with the tipping bucket device (Fig 1).

**Fig 2:**
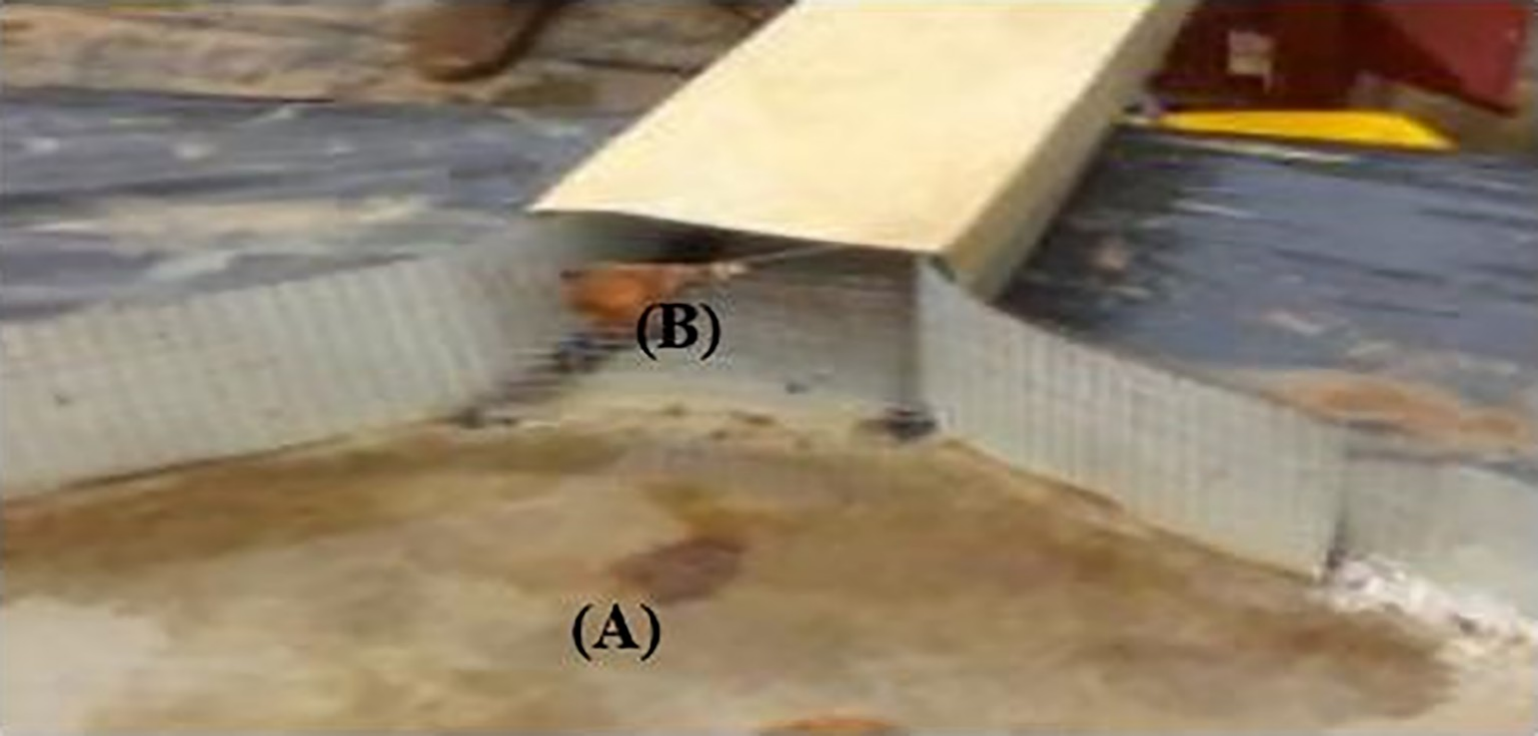
Collecting trough with aluzinc sheet at the end of each runoff plot and the mesh fixed between the channel and the collecting trough to retain the first portion of the runoff loads.

The tipping bucket device consisted of a collecting trough, tipping bucket and counter as described below:

***Collecting trough***: After the last row of crops, there was trapezoidal surface (covered by aluzinc sheets) to retain the first portion of sediments (which were analyzed separately for the nutrients content) from the plot whilst the rest of the water and the loads were passed through a mesh of 0.1 cm diameter for collection with the tipping bucket (Fig 2).

***Tipping bucket devices and counter***: After the mesh, the rest of water and its loads were passed through a channel of diameter 22.5 cm, ending into a tipping bucket with two specific buckets (sides) with a known tipping volume for each (Fig 1). Once a bucket was filled with water or at the tipping volume, it tipped automatically and this was recorded from a counter fixed to the system. The number recorded from the counter multiplied by the tipping volume of each bucket gave the volume of runoff collected from each plot.

The sampling was done every time after an erosive rainfall. A 500 mL sample was taken from the total runoff for nutrient quantification. Also, from the direct sediment, a sub-sample of 250 g was taken and air-dried for nutrient loss determination through the sediments.

### 2.4. Nutrient loss

During erosion process, plant nutrients are transported in runoff and sediments. The surface layers are the most affected and where most of soil nutrients for plant nutrition are concentrated [9]. To assess the nutrient loss (Eq 1), samples of runoff (100 ml) and sediment (100 g) were taken from the total runoff and direct sediment (retained on the collecting trough) respectively from the collecting trough fitted to each treatment plot.

$$Total\;amount\;of\;nutrient\;lost\;\left(N\right)=N_{1}+N_{2}$$ (1)

where:

N1 = Nutrient loss through the runoff,

N2 = Nutrient loss through sediment.

#### 2.4.1. Nutrient loss through the runoff (N_1_)

Nutrients concentration in the runoff, N_1_ was computed using Eq (2).

$$N1=n_{1}\;*\;Rt$$ (2)

Where: N_1_ (g) = Total amount of each nutrient lost through runoff;

Rt (L) = the total amount of runoff measured in situ and

n_1 (_g/L_)_ = concentration of each element in the runoff determined as described under section 2.7.

#### 2.4.2. Nutrient loss through sediment (N_2_)

The amount of each nutrient lost through the sediment was determined using Eq (3).

$$N_{2}=n_{2}\;*\;S_{2}$$ (3)

where: N_2_ (g) = the total amount of each nutrient lost in the sediment collected on the trough;

S_2 (_g) = the total amount of direct soil sediment collected on the trough;

n_2 (_g g^-1^) = the concentration of each nutrient in the sediment determined as described under section 2.7.

### 2.5. Enrichment ratio (ER)

Soil erosion does not affect only the site where it originates but also the soil and the ecosystems outside the eroded area. This is expressed as the accumulation of sediments and nutrients on the new site of deposition with negtaive impacts on plants and other living organims as well as soil properties. The eroded materials are generally richer in plant nutrients and fine soil particles compared to the remaning soil on the eroded site and this is defined by enrichment ratio (ER) (Eq 4) [12,18]. Enrichment ratio greater than one indicates that the sediment is richer in nutrienets than the parent soil.

$$\mathrm{ER}=\frac{\mathrm{Nutrient}\;\mathrm{concentration}\;\mathrm{in}\;\mathrm{sediment}\;(g\;kg^{-1})}{\mathrm{Nutrient}\;\mathrm{concentration}\;\mathrm{in}\;\mathrm{parent}\;\mathrm{soil}\;\left(g\;kg^{-1}\right)}$$ (4)

### 2.6. Economic value of the different nutrients lost through runoff and sediment

In this study, the replacement cost method was used to estimate the cost of fertility erosion. This involved converting nutrient loss to existing fertilizer forms to assess the monetary value of the nutrients lost through erosion [19] under the different soil and crop management practices. The inorganic fertilizers applied were: urea, TSP and KCl with the concentration of 46%, 46% and 60% for N, P_2_O_5_ and K_2_O respectively. Therefore, the three macronutrients (N, P, and K) analyzed from the runoff and sediment were converted into monetary values based on the three straight inorganic fertilizers (Urea, TSP and KCl) which were applied under the different cropping systems using the factors of 0.44 and 0.8 for converting P and K to P_2_O_5_ and K_2_O respectively.

Using the concentration of active ingredient in each fertilizer indicated above, it was possible to determine the monetary value of soil nutrients lost through erosion under each treatment. The prevailing market price in Ghana cedis) of each fertilizer was used to compute monetary value of soil fertility erosion. The local currency (Ghana cedis) was converted into US dollars after each growing season.

### 2.7. Laboratory analysis

For sediment analyses, total nitrogen (TN), available phosphorus (P) and exchangeable potassium (K) were determined using the methods described by [18]. The total nitrogen content of the dry soil was determined using the Kjeldahl digestion and distillation procedure. Phosphorus was determined using the Bray P1 method whilst exchangeable potassium was determined by the flame photometry procedure.

With respect to runoff analysis, 10 mL runoff/water sample was measured and transferred into a 500 mL digestion flask and the N content determined by the Kjeldahl process [20]. Phosphorus and potassium concentrations in the runoff were analyzed from a thoroughly mixed and homogenized sample (50 mL) to which 30 mL and 10 mL of HCl and HNO_3,_ respectively were added and digested. Phosphorus and the potassium concentrations were then determined by Bray I and flame photometry methods respectively. Soil particle size analysis was done for eroded sediments and the soil remaining on the site using the hydrometer method [20]. All analyses were performed in the Soil Science Laboratory of the Department of Crop and soil Sciences of the Kwame Nkrumah University of Science and Technology, Kumasi.

### 2.8. Statistical analysis

Analysis of variance was conducted to determine the effects of (and interaction between) cropping systems and soil amendments on soil nutrient loss characteristics as well as monetary cost of erosion. Prior to analysis of variance, the data was checked for normality using residual plots in GENSTAT v.12. Treatment means were compared using the least significant difference (LSD) method at 5%.

## 3 Results

### 3.1 Cumulative soil nutrient loss through erosion during three Consecutive Cropping seasons

The cumulative amount of nutrients lost under the different cropping systems and soil amendments are presented in Table 1. The nutrients assessed were the N, P, and K, which were applied via chemical fertilizers in combination with biochar.

**Table 1:** Cumulative soil nutrient loss through erosion during three consecutive cropping seasons.

Treatments	N (kg ha^-1^)	P (kg ha^-1^)	K (kg ha^-1^)
**Cropping systems (CS)**			
Cowpea (CW)	12.38	6.67	5.81
Maize (MZ)	19.71	8.12	7.27
Soybean (SB)	16.75	6.81	6.61
Maize+Soybean (MZ+SB)	17.12	7.49	6.75
CV (%)	11.9	18.1	11.2
LSD (5%)	5.23	1.08	1.30
**Soil amendments (SA)**			
Control	20.43	8.42	7.87
Biochar (BC)	18.83	6.82	6.47
Inorganic fertilizer (NPK)	15.33	5.78	6.15
NPK+BC	14.16	5.58	5.94
CV (%)	15.1	12.7	12.0
LSD (5%)	4.44	0.64	0.68
**CS x SA**			
MZ x Control	22.45	9.55	8.15
MZ x BC	19.08	7.16	7.00
MZ x NPK	16.49	6.93	6.05
MZ x NPK+BC	14.60	6.80	5.82
M Z+SB x Control	21.13	8.82	7.92
MZ+SB x BC	19.08	7.92	7.24
M Z+SB x NPK	17.56	8.12	6.12
MZ+SB x NPK+BC	16.08	7.63	5.42
SB x control	20.80	7.32	7.79
SB x BC	20.56	7.05	6.40
SB x NPK	16.83	6.17	6.08
SB x NPK+BC	14.04	6.68	5.86
CW x Control	17.31	6.54	7.34
CW x BC	18.48	6.24	5.47
CW x NPK	14.48	5.65	5.98
CW x NPK+BC	13.89	5.04	5.17
CV (%)	12.4	17.9	14.9
LSD (5%)	6.88	1.44	1.63

CV: Coefficient of Variation; LSD: Least Significant Difference

The cropping systems, soil amendments and their interactions showed significant differences (p <0.05) in N, P, K eroded at the end of all three cropping seasons. Among the cropping systems evaluated, sole maize was the most sensitive to fertility erosion with the highest amounts of N, P and K losses (19.71; 8.12 and 7.27 kg ha^-1^ respectively) while sole cowpea had the lowest values (12.38; 6.67 and 5.81 kg ha^-1^) for all three nutrient elements. The highest nutrient loss was observed on the control plots and the least on plots, which received external inputs especially the inorganic fertilizer treatments associated with biochar. The respective average values of N, P and K were 20.43; 8.42 and 7.87 kg ha^-1^ for the control plots and 14.15; 5.58 and 5.94 kg ha^-1^ for NPK + biochar amended plots.

**Fig 3:**
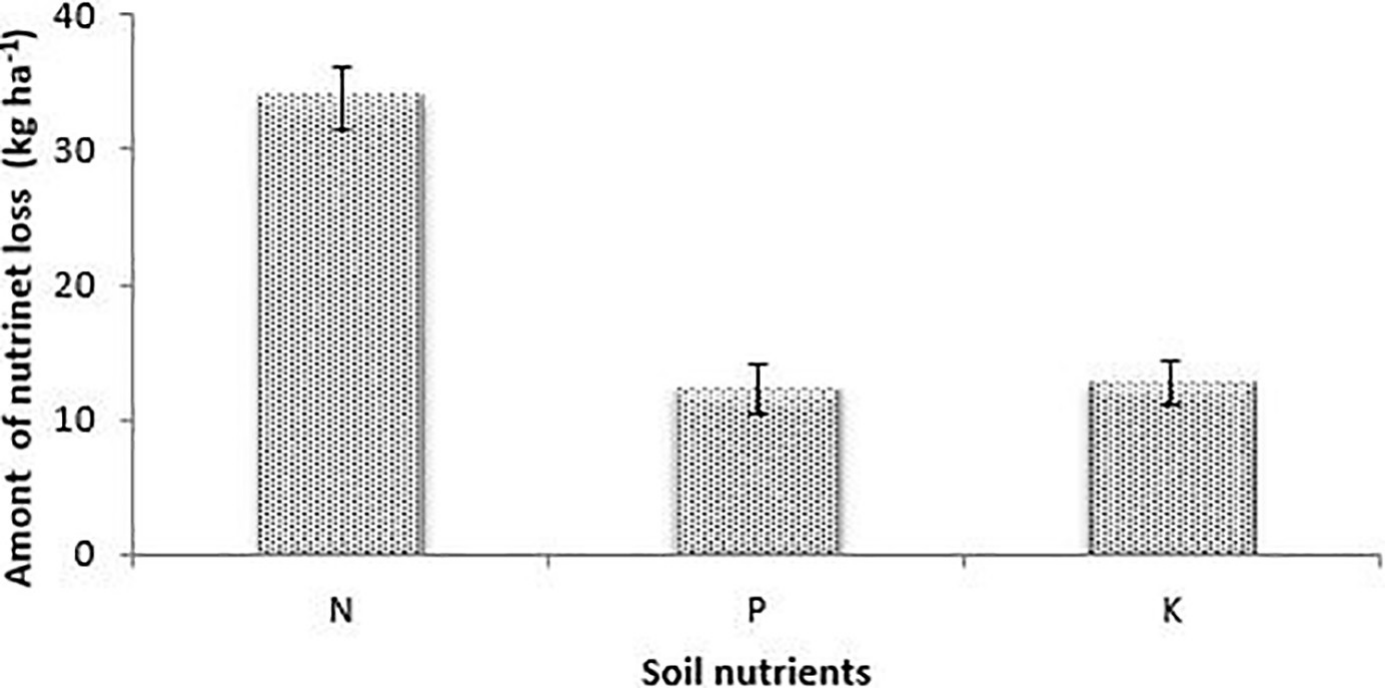
Cumulative soil nutrient loss on bare plots.

For the interaction effect, each cropping system without any external amendment produced the highest nutrient loss whilst the lowest were observed under cropping systems associated with inorganic inputs (Table 1). The bare plots had the highest nutrient loss compared to all the cropped plots (Fig 3).

### 3.2 Enrichment ratio of the soil particles and nutrients eroded under the different soil amendments and cropping systems

**Fig 4:**
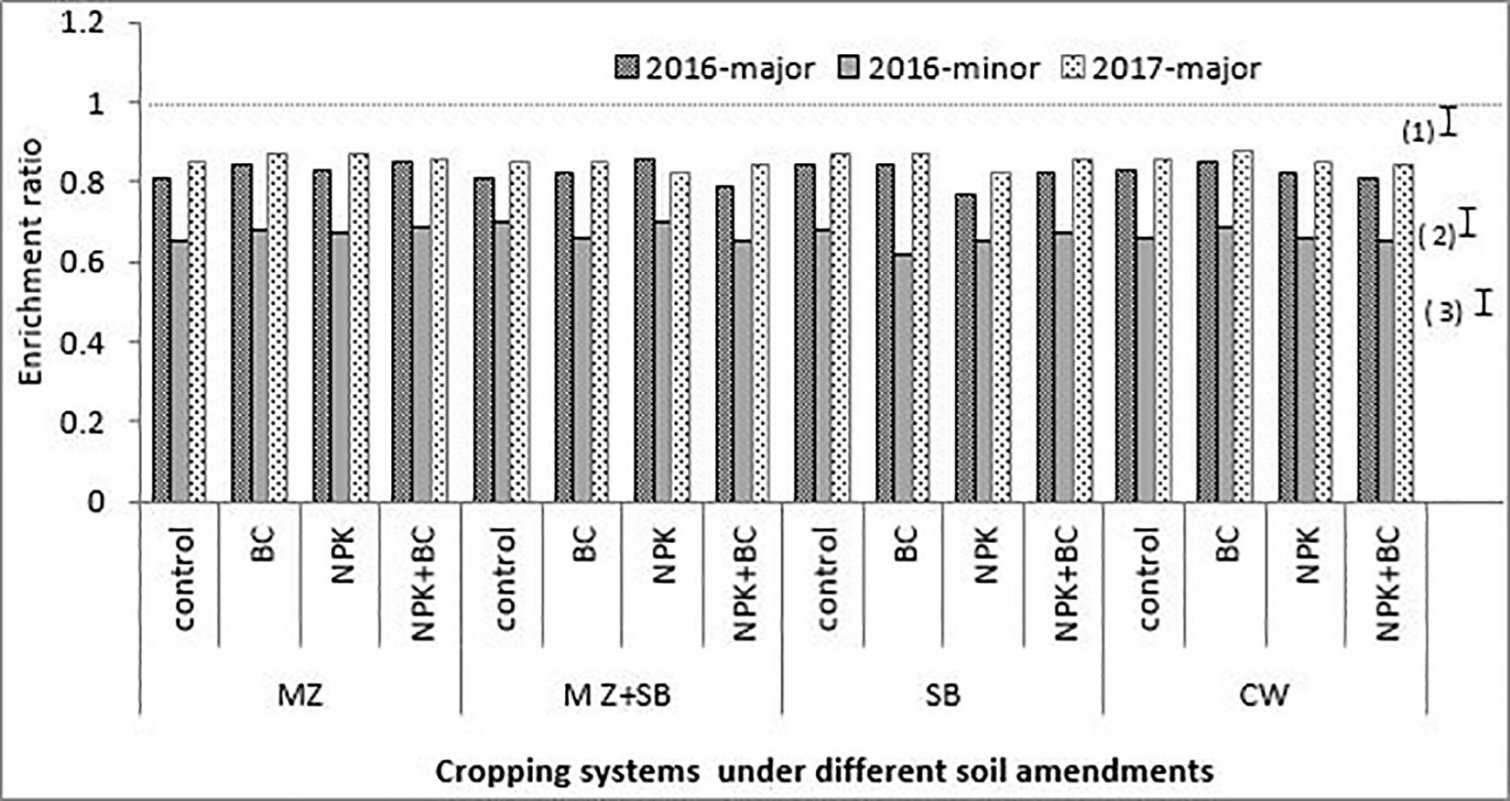
Sand enrichment ratio during the three cropping seasons. The bars (1), (2) and (3) are LSD (5%) for 2016-major, 2016-minor and 2017-major 2017 Seasons, respectively, MZ = sole maize, SB = sole soybean, CW = sole cowpea and MZ+SB = maize and soybean intercrop.

**Fig 5:**
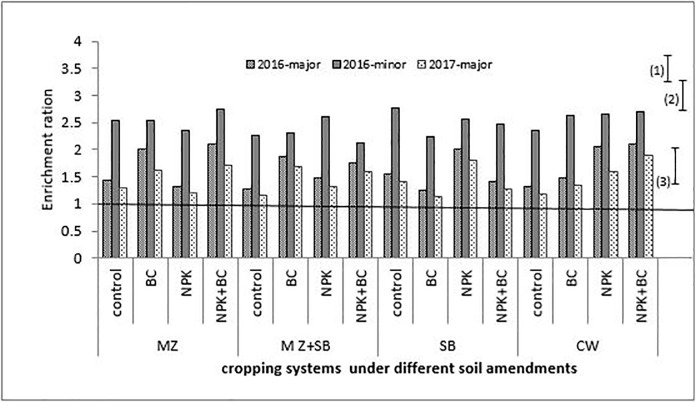
Silt enrichment ratio during the three cropping season. The bars (1), (2) and (3) are LSD (5%) for 2016-major, 2016-minor and 2017-major seasons, respectively; MZ = sole maize, SB = sole soybean, CW = sole cowpea and MZ+SB = maize and soybean intercrop.

**Fig 6:**
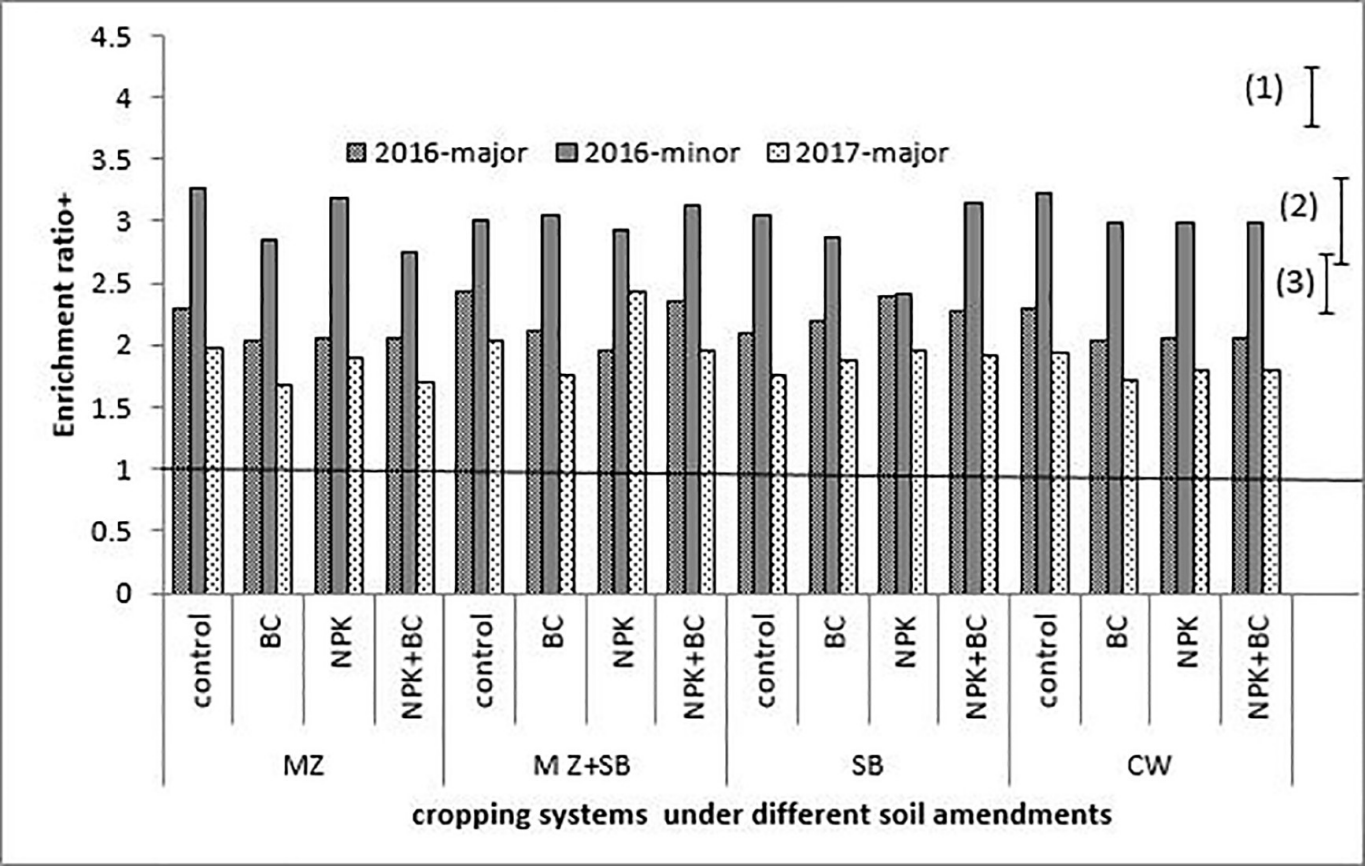
Clay Enrichment ratio during the three-cropping seasons. The bars (1), (2) and (3) are LSD (5%) for 2016-major 2016-minor and 2017-major seasons, respectively; MZ = sole maize, SB = sole soybean, CW = sole cowpea and MZ+SB = maize and soybean intercropped.

Tables 2, 3 and 4 and, Figs 4, 5 and 6 represent the different ERs for the selected soil properties during the three cropping seasons. The chemical parameters (N, P and K) had ER greater than 1 for the individual factors and their interactions. In general, for all the crop nutrients, the ERs were higher during the minor seaon than in the major seasons for all plots with inorganic soil amendments. Moreover, all the amended plots had slightly higher ERs than the unamended plots.

**Table 2:** Effect of soil amendments, cropping systems and their interactions on nitrogen enrichment ratio.

Treatments	N enrichment ratio		
**Cropping systems (CS)**	**2016- major**	**2016-minor**	**2017-major**
Cowpea (CW)	1.54	2.20	1.30
Maize (MZ)	1.85	2.91	1.60
Soybean (SB)	1.79	2.44	1.72
Maize+Soybean (MZ+SB)	1.58	2.63	1.57
CV (%)	2.8	7.30	6.70
LSD (5%)	0.16	0.4	0.15
**Soil Amendements (SA)**	**2016- major**	**2016-minor**	**2017-major**
Control	1.73	1.46	1.59
Biochar (BC)	1.56	1.85	1.45
Inorganic fertilizer (NPK)	1.86	3.10	1.69
NPK+BC	1.62	3.09	1.47
CV (%)	4.9	7.6	5.00
LSD (5%)	0.45	0.64	0.38
**CS x SA**	**2016- major**	**2016-minor**	**2017-major**
MZ x Control	1.60	2.06	1.37
MZ BC	1.65	2.45	1.41
MZ x NPK	2.22	3.74	1.90
MZ x NPK+BC	1.94	3.44	1.72
M Z+SB x Control	1.53	1.31	1.51
MZ+SB x BC	1.82	3.33	1.81
M Z+SB x NPK	2.32	3.04	1.79
MZ+SB x NPK+BC	1.95	2.81	1.19
SB x control	1.31	1.33	1.32
SB x BC	1.35	2.27	1.38
SB x NPK	1.94	2.75	1.81
SB x NPK+BC	1.73	3.43	1.53
CW x Control	1.60	1.11	1.33
CW x BC	1.43	2.20	1.19
CW x NPK	1.93	2.86	1.27
CW x NPK+BC	1.79	2.65	1.40
CV (%)	11.7	19.9	9.5
LSD (5%)	0.79	1.15	0.67

CV: Coefficient of Variation; LSD: Least Significant Difference

**Table 3:** Effect of soil amendments, cropping systems and their interactions on available phosphorus enrichment ratio.

Treatments	P enrichment ratio		
**Cropping systems (CS)**	**2016- major**	**2016-minor**	**2017-major**
Cowpea (CW)	1.64	2.38	1.39
Maize (MZ)	2.00	1.95	1.79
Soybean (SB)	1.44	2.63	1.40
Maize+Soybean (MZ+SB)	1.46	2.09	1.48
CV (%)	17.9	14.4	17.8
LSD (5%)	0.87	1.08	0.8
**Soil Amendements (SA)**	**2016- major**	**2016-minor**	**2017-major**
Control	1.55	1.58	1.33
Biochar (BC)	1.39	1.96	1.18
Inorganic fertilizer (NPK)	1.68	2.94	1.84
NPK+BC	1.67	2.58	1.70
CV (%)	19.9	12.2	16.5
LSD (5%)	0.61	0.58	0.58
**CS x SA**	**2016- major**	**2016-minor**	**2017-major**
MZ x Control	1.35	1.44	1.67
MZ BC	1.46	1.98	1.25
MZ x NPK	2.43	2.50	1.78
MZ x NPK+BC	2.18	1.94	1.98
M Z+SB x Control	1.40	1.78	1.38
MZ+SB x BC	1.32	1.63	1.31
M Z+SB x NPK	1.60	2.26	1.63
MZ+SB x NPK+BC	1.51	2.67	1.58
SB x control	1.23	1.70	1.24
SB x BC	1.13	2.35	1.16
SB x NPK	1.85	3.14	1.84
SB x NPK+BC	1.54	3.37	1.38
CW x Control	1.21	1.44	1.01
CW x BC	1.22	1.87	1.01
CW x NPK	1.93	3.88	1.66
CW x NPK+BC	2.22	2.33	1.88
CV (%)	19.3	14	15.9
LSD (5%)	1.12	1.16	1.21

CV: Coefficient of Variation; LSD: Least Significant Difference

**Table 4:** Effect of soil amendments, cropping systems and their interactions on potassium enrichment ratio.

Treatments	K enrichment ratio		
**Cropping systems (CS)**	**2016- major**	**2016-minor**	**2017-major**
Cowpea (CW)	1.65	1.86	1.38
Maize (MZ)	2.36	2.64	2.15
Soybean (SB)	1.84	2.06	1.75
Maize+Soybean (MZ+SB)	2.18	2.17	2.15
CV (%)	20.8	12.2	13.0
LSD (5%)	0.53	0.44	0.46
**Soil Amendements (SA)**	**2016- major**	**2016-minor**	**2017-major**
Control	1.85	1.51	1.69
Biochar (BC)	2.03	1.67	1.88
Inorganic fertilizer (NPK)	2.17	3.06	1.98
NPK+BC	1.98	2.48	1.93
CV (%)	14.1	21.5	12.9
LSD (5%)	0.21	0.30	0.36
**CS x SA**	**2016- major**	**2016-minor**	**2017-major**
MZ x Control	1.95	1.53	1.66
MZ BC	2.11	2.23	1.89
MZ x NPK	2.88	3.67	2.12
MZ x NPK+BC	2.60	3.13	2.17
M Z+SB x Control	2.15	1.79	2.08
MZ+SB x BC	2.02	1.45	1.95
M Z+SB x NPK	2.39	3.02	2.28
MZ+SB x NPK+BC	2.14	2.41	2.23
SB x control	1.60	1.48	1.54
SB x BC	2.21.	1.48	2.18
SB x NPK	1.82	2.96	1.79
SB x NPK+BC	1.71	2.30	1.64
CW x Control	1.72	1.24	1.42
CW x BC	1.76	1.54	1.56
CW x NPK	1.98	2.58	1.77
CW x NPK+BC	1.88	2.10	1.73
CV (%)	14.1	18.3	13.9
LSD (5%)	0.84	1.44	0.74

CV: Coefficient of Variation; LSD: Least Significant Difference

During the cropping seasons, clay and silt particles had high ERs (greater than unity) with higher values in the minor season than in the major seasons. The sand particles had ERs less than unity for all the three growing seaons which were slightly higher in the major season than in the minor season (Fig 4).

### 3.3 Economic value of nutrients lost due to soil erosion

The monetary values of soil nutrient loss under the different soil amendments and cropping systems in Ghana (for each season and cumulatively) are presented in Table 5. Indeed, the highest cumulative monetary values of soil nutrients lost through erosion were observed on the control plots with the least on plots treated with biochar + inorganic fertilizers under all cropping systems throughout the study period. Higher values were recorded in the major seasons than in the minor rainy season. Under all the cropping systems, the inorganic fertilizers treated plots had the lowest monetary values of soil nutrient loss compared to sole biochar and control plots. In general, legume-based cropping systems were the most economically viable compared to the maize based systems in terms of the monetary value of soil nutrients lost.

**Table 5:** Monetary values of the primary macronutrients lost under different cropping systems and soil amendments.

	Economic nutrient loss (US$ ha^-1^ season)			
Amendments X Cropping systems	2016- major	2016-minor	2017-major	Cumulative (3 seasons)
NPK+BC x SB	15.42	10.83	12.38	38.32
NPK x SB	17.22	12.67	14.49	44.58
BC x SB	20.94	12.27	14.03	47.64
Control x SB	22.20	15.30	19.49	56.99
NPK+BC X MZ+ SB	17.64	13.60	15.54	46.02
NPK X MZ+ SB	17.87	13.17	17.34	48.19
BC X MZ+ SB	16.98	12.95	17.09	47.01
Control X MZ+ SB	19.40	15.53	22.17	57.10
NPK+BC X MZ	16.85	13.67	15.63	46.75
NPK X MZ	16.67	15.22	17.40	48.29
BC X MZ	24.20	15.75	18.00	57.85
Control X MZ	24.41	19.98	22.83	67.21
NPK+BC X CW	9.29	10.05	11.49	30.62
NPK X CW	17.05	10.02	11.46	36.50
BC X CW	16.32	12.17	13.91	42.40
Control X CW	18.48	17.30	17.86	53.63
LSD (5%)	2.83	2.43	3.21	6.12
CV (%)	12.5	17.4	15.1	14.3

MZ = sole maize, SB = sole soybean, CW = sole cowpea and MZ+SB = maize and soybean intercropped

CV: Coefficient of Variation; LSD: Least Significant Difference; NPK = Nitrogen+ Phosphorus+ Potassium; BC = Biochar

Sole maize had higher values compared to the intercropping system, which was slightly higher than the sole soybean. In general, sole cowpea had the least economic loss from soil erosion than all the other cropping systems evaluated.

With respect to the interaction between the soil amendments and cropping systems, the cumulative values ranged from 30.82 to 67.21 US$ ha^-1^ for Cowpea x NPK and sole maize x Control, respectively (Table 5). The specific economic loss observed under the different treatments is normally related to their ability to reduce soil erosion and nutrient transport through runoff and sediments during the growing season.

## 4. Discussion

### 4.1 Soil nutrient loss through erosion during three consecutive cropping seasons

The relatively lower amounts of nutrient loss observed under sole cowpea cropping system (Table 1) could be explained by the least soil loss under this treatment. The bare plots, due to the absence of land cover and its attendant soil physical degradation, were more affected by nutrient loss compared to plots under crop management (Fig 3 and Table 1). Poor land cover increases soil erosion through physical and mechanical impact of rainfall and aggregate destruction [21]. This, subsequently, leads to soil surface sealing and decreased infiltration with increased runoff and soil loss [21].

Biochar with its effect on soil physical improvement [22] and soil nutrient stability, resulted in lower nutrient losses (Table 2) which is essential for sustainable cropping and environmental protection. We infer some mechanisms to be involved. First, biochar is known to function as a binding agent connecting soil microaggregates into macroaggregates. Also, under acidic environments, its oxidized surface which includes hydroxyl and carboxylic groups adsorbs soil particles and clays to form macroaggregates [23]. It also fixes some soil nutrients useful for direct crop nutrition from slow or progressive release [24, 25]. This fixation reduces the direct nutrient loss through erosion [17]. In our study, biochar and NPK fertilizer interactions produced the least cumulative loss for all three nutrients assessed, suggesting a strong basis for its incorporation into soil management practices to reduce fertility erosion in SSA.

Despite the high concentration of soil nutrients in the sediments and runoff, soil erosion was strongly reduced under nutrient addition (Table 1). This is due to the fact that soil nutrient application via external inputs improves crop performance through increased above and underground biomass which reduces runoff velocity [26]. In a study carried out in sorghum based cropping systems in Burkina Faso [22] found also that nutrient loss was lower on plots that received urea fertilizer compared to control plots.

With respect to cropping systems, the higher amounts of N, P and K lost under sole maize (Table 1) was related to less land cover with increased soil sediment transport. Conversely, with its good land cover, sole cowpea cropping system had the least amounts of nutrient loss, emphasizing the role of legumes in soil nutrient conservation on croplands.

### 4.2 Enrichment ratios under soil amendments and cropping systems

All nutrients assessed during the cropping seasons, had ERs greater than 1, showing the ability of soil erosion to transport the most fertile soil layers out of cropped area. The higher ERs of soil nutrients observed in the minor season (Tables 2, 3 and 4) was due to low soil moisture content based on rainfall amount.s with low nutrient solubility [8] probably leading to increase in nutrients concentration in the runoff and sedimens. The detached top-layers are highly concentrated in soil nutrients [7] and this strongly compromizes agricultural activities due to acute nutrient depletion in eroded soils. Under high rates of runoff and sediment, nutrient dilution is high [27] and this should be expressed by lower ERs of nutrients as per our observations in the major seasons. Although the total amounts of nutrient loss was higher on the unamended plots than treated plots (Table 1), the latter generally, had higher ERs under the different cropping systems (Tables 2, 3 and 4) indicating that nutrients supplied from the different amendments were washed away, being highly concentrated in the runoff and sediments.

The soil particles during the erosion process, exhibited different degrees of erodibility with respect to the erosive factors. The ER > 1 observed for the clay and silt particles (Figs 5 and 6) is an indication that eroded materials were richer in fine particles. Due to the selectivity nature of the process, the fine soil particles, which are richer in plant nutrients, were the most eroded. Generally, soil sediments contain higher amounts of soil nutrients in available forms than the soil from which it is eroded [9,28]. This nothwithstanding, the higher ERs observed for sand during the two major seasons compared to the minor season, was probaly due to the storms characteristics.

Plots treated with inorganic fertilizers had generally higher ERs compared to those with sole organic amendment showing that fertilizers applied on erodible lands may be lost through runoff and sediment and increase off-site effects (e.g. eutrophication of water bodies) with nutrients accumulations. However, common cropping systems in smallholder farming systems asscociated with biochar addition are advisable to reduce the impact of these losses.

### 4.3 Economic value of soil nutrient loss due to erosion

Globally, due to soil erosion, the annual amount of fertilizers mobilized is equivalent to 34 US$ billion for N and 80 US$ billion for P which is an important financial loss; while the global agricultural food production is valued at US$ 4000 billion [3]. One of the objectives of soil amendments is to restore the different nutrients lost through different pathways (e.g., plant up take, soil erosion). This may be achieved through application of inorganic fertilizers from the markets.

Nutrient loss through runoff and sediment transport converted into monetary values showed that beyond direct crop nutrition effect [27], soil nutrient management is an important component for sustainability of agriculture. The reduced monetary values in terms of nutrient loss observed under the soil amendments and each cropping system (Table 5), explained the effect of fertilizers application on soil erosion management. Under poor soil and crop management, the rate of soil and nutrient losses are very high. In our study, the higher cumulative monetary values observed for the control plots were related to the considerable amounts of soil and runoff losses on these plots. Indeed, the lower cumulative economic loss observed on plots treated with inorganic fertilizers associated with biochar ranging from 30.62 to 46.75 US$ ha^-1^ against 53.62 to 57.10 US$ ha^-1^ observed on the control plots (without nutrient addition) was, due to soil erosion reduction under the former. Moreover, the low values observed under NPK+BC accord with the low amounts of nutrient loss (Table 1) and ERs (Tables 2, 3 and 4) observed under this treatment. Although the economic losses were below the cost of the nutrients applied (102.17 US$ ha^-1^ for NPK), soil erosion remains an important constraint to sustainable nutrient management in crop intensification systems. Though a small quantity of nutrient is lost seasonally, its long-term effect on crop production is costlier than the seasonal soil management to reduce the degradation. This calls for effective measures to reduce losses as shown in this study where cumulative economic loss under sole cowpea treated with NPK + BC was the least (Table 5). Integrated management of biochar and mineral fertilizers under cowpea based cropping systems therefore holds promise to reducing economic loss through soil erosion on arable lands in SSA.

The economic value of nutrient loss on the control plot was higher due to the magnitude of soil and runoff losses from these unmanaged plots as alluded to earlier. Moreover, the amounts of fertilizer applied were not high enough to increase soil nutrient entrainment into the sediment and runoff compared to the important amount of soil lost from these plots. Therefore, the total amount of nutrient lost due to erosion is mostly related to the amount of soil loss than to nutrient application. A study carried out by [9] showed that the economic value for NPK plots under maize was highly reduced compared to the plots without any amendments and the bare plots.

The economic value of soil erosion was based on soil fertility erosion using the cost replacement method [9]. However, even though the method gives the magnitude of erosion on nutrient loss, it presents some limitations: soil erosion affects other nutrients and other forms of soil degradation, which may require investments for restoration. Also, eroded nutrient forms and the nutrient forms in the fertilizers may be slightly different for accurate conversion. This notwithstanding, the method is still reliable to assess the economic value of soil loss under soil erosion constraints [19].

## 5. Conclusion

Soil erosion based on nutrient loss characteristics were, influenced by cropping systems and soil amendments. Nutrient management practices showed positive effect on soil and nutrient loss reduction which was lower under sole inorganic fertilizers or in combination with biochar under different cropping systems, especially the sole cowpea system. Cropping systems associated with soil nutrient addition are therefore multipurpose methods to improve crop production as well soil nutrient loss reduction.

Combined application of biochar and mineral fertilizers reduced ERs of nutrients. Soil nutrient loss was, more important during the major seasons as a result of high rainfall amounts. Fine soil particles (clay and silt) had higher ERs than sand particles.

Monetary value of nutrient loss was affected by the different management practices imposed. Cropping systems without any amendment suffered more economic loss due to nutrient loss. Soil amendments under legume-based cropping systems reduced soil nutrient loss with least economic loss. These findings give a new opportunity to highlight the importance of sustainable crop management to reduce nutrient loss on croplands in SSA.

## Supporting information

S1 Table Sand Enrichment ratio. MZ = sole maize, SB = sole soybean, CW = sole cowpea and MZ+SB = maize and soybean intercropped.
(XLSX)

S2 Table Clay Enrichment ratio. MZ = sole maize, SB = sole soybean, CW = sole cowpea and MZ+SB = maize and soybean intercropped.
(XLSX)

S3 Table Silt Enrichment ratio. MZ = sole maize, SB = sole soybean, CW = sole cowpea and MZ+SB = maize and soybean intercropped.
(XLSX)

S4 Table Nutrient Enrichment ratio. MZ = sole maize, SB = sole soybean, CW = sole cowpea and MZ+SB = maize and soybean intercropped.
(XLSX)

S5 Table Total amount of nutrient loss. MZ = sole maize, SB = sole soybean, CW = sole cowpea and MZ+SB = maize and soybean intercropped.
(XLSX)
